# Long‐Term Treatment Patterns in Norwegian ADHD‐Medicine Users: A Cohort Register‐Based Study With 5 Years of Follow‐Up, 2012–2021

**DOI:** 10.1002/pds.70486

**Published:** 2026-10-12

**Authors:** Espen Anker, Anne Halmøy, Mats Fredriksen

**Affiliations:** ^1^ Klinikk Toppetasjen Oslo Norway; ^2^ Division of Psychiatry Haukeland University Bergen Norway; ^3^ Department of Clinical Medicine University of Bergen Bergen Norway; ^4^ Division of Mental Health and Addiction Vestfold Hospital Trust Tonsberg Norway

**Keywords:** attention‐deficit/hyperactivity disorder, methylphenidate, persistence, pharmacoepidemiology, prescription registry, treatment switching

## Abstract

**Purpose:**

To improve interpretation of long‐term ADHD medication treatment trajectories in Norway by examining sex‐ and age‐specific persistence, distinguishing within‐substance product switching from active‐ingredient (ATC substance) switching, and describing the medication and prescriber context of the last observed dispensation before non‐persistence.

**Methods:**

This study used population‐based data from the Norwegian Prescription Database (NorPD) to identify 33 706 incident users initiating ADHD medication in 2012–2016, defined using an individual‐specific 5‐year look‐back. Participants were followed for up to 5 years until 2021. We estimated dispensing‐based persistence, defined non‐persistence as the first dispensing gap of ≥ 396 days/13 months, characterised switching as either within‐substance product switching or active‐ingredient (ATC substance) switching, and described the last dispensed medication and prescriber specialty recorded before non‐persistence.

**Results:**

The cohort included 19 898 men (59%) and 13 808 women (41%); 97%–98% initiated methylphenidate. Five‐year persistence was 39% in men and 35% in women. During follow‐up, 41% had no switch and 26% had ≥ 4 switches. Among first switches, within‐substance product switching predominated (80.9% in men; 77.5% in women), while active‐ingredient switching was less common (19.1% and 22.5%, respectively). Among those who met the non‐persistence definition during follow‐up (62.4%), the last dispensed medication was methylphenidate (76%), atomoxetine/guanfacine (12%), lisdexamfetamine (7%), and dexamphetamine (3%), respectively; the last prescriber specialty recorded on the last dispensation was a general practitioner for 51.9% and a specialist for 48.1%.

**Conclusions:**

In Norway, most incident ADHD medication users met a dispensing‐based definition of non‐persistence within 5 years. Switching was frequent, but most first switches occurred within the same active substance rather than between substances, suggesting that many observed changes in dispensing data may reflect product substitution or formulation change rather than a true change in pharmacological strategy. These findings may improve interpretation of long‐term treatment trajectories.

## Key Points

- In this nationwide cohort of incident users (2012–2016), 5‐year persistence was 39% in men and 35% in women using a dispensing‐gap definition of non‐persistence (≥ 396 days).
- Medication switching was common: 26% experienced ≥ 4 product switches within 5 years. Most first switches were within‐substance product switches (same ATC substance): 80.9% in men and 77.5% in women, whereas active‐ingredient (new ATC substance) switches were a minority (19%–23%) and were concentrated in a few pathways, mainly methylphenidate → lisdexamfetamine or atomoxetine.
- Among those who met the non‐persistence definition, methylphenidate was the last dispensed medication in 76%, and the last prescriber specialty recorded was a general practitioner in 51.9%.
- Product switching within the same ATC substance may reflect substitution and reimbursement/price mechanisms rather than intentional selection of a more effective medicine.

## Plain Language Summary

Using nationwide prescription data from Norway, we followed 33 706 people who started ADHD medication and examined their treatment patterns for up to 5 years. Most users did not continue redeeming ADHD medication continuously throughout follow‐up, and persistence after 5 years was 39% in men and 35% in women. Treatment duration was shortest in adolescents and young adults. Medication switching was common, but most first switches were between products containing the same active substance rather than between different active substances. This suggests that many apparent switches in prescription data may reflect generic substitution or formulation changes rather than a true change in treatment strategy. Among individuals who later met the definition of non‐persistence, methylphenidate was most often the last dispensed medication, and the last recorded prescriber was almost equally often a general practitioner or a specialist. These findings provide a more detailed picture of long‐term ADHD medication use in Norway and may improve interpretation of treatment trajectories in prescription registry data.

## 1 Introduction

Attention‐deficit/hyperactivity disorder (ADHD) is a common neurodevelopmental condition characterised by persistent patterns of inattention, hyperactivity, and impulsivity that interfere with daily functioning [1]. Although initially conceptualised as a childhood disorder, ADHD frequently persists into adolescence and adulthood, affecting 2%–3% of adults globally [2]. Pharmacological treatment is a cornerstone of ADHD management and is associated with improved symptom control, social functioning, and academic outcomes [3, 4].

During the past two decades, use of ADHD medications has increased markedly in many settings and may parallel increasing diagnosis or recognition of ADHD [5, 6]. A multinational pharmacoepidemiological analysis covering 13 countries showed increasing ADHD medication use over time [6]. In Norway, the prevalence of ADHD medication users aged 6–64 years rose from 5.2 per 1000 in 2006 to 19.4 per 1000 in 2022 [7]. However, detailed descriptions of product‐level switching and the prescribing context around discontinuation remain limited.

International studies consistently report low persistence and high discontinuation rates. In a large cross‐national study, Brikell et al. [8] found that only 30%–40% of individuals still redeemed medication 5 years after initiation. Reported reasons for discontinuation include reduced symptoms or perceived need for medication, adverse effects, perceived lack of benefit, adherence challenges, and changes in follow‐up arrangements [9, 10]. These patterns are especially pronounced during adolescence and early adulthood, coinciding with life transitions and challenges in continuity of care [8, 11].

### 1.1 The Norwegian Context

In Norway, ADHD pharmacotherapy is regulated under a universal healthcare system. Psychiatrists must initiate stimulant treatment, but once the patient is clinically stable, the general practitioner (GP) may continue prescribing under supervision [12]. For adults, only intermediate‐acting methylphenidate formulations are approved as first‐line treatments, while lisdexamfetamine may be prescribed when methylphenidate has provided insufficient symptom control or caused unacceptable adverse effects. Atomoxetine is not pre‐approved for reimbursement for adult patients, and guanfacine has not approved ADHD as an indication for adults, limiting their use in this age group. For children, both short‐, intermediate and long‐acting methylphenidate preparations and dexamphetamine including lisdexamphetamine are authorised, together with atomoxetine and guanfacine. Numerous generics have entered the market and potentially contribute to frequent switching. All these different medicines have been accessible in Norway since before 2005 except lisdexamphetamine and guanfacine that both were introduced in 2015.

Norwegian dispensing data were included in the multinational study by Brikell et al. [8] which reported country‐specific Kaplan–Meier estimates of persistence and discontinuation in the 5 years after initiation, stratified by age groups at initiation, but not by sex.

However, analyses designed primarily for cross‐national comparability provide limited details on the treatment trajectories underlying these estimates within a national setting. In particular, register‐based studies may group medication changes broadly as “switching”, although changes observed in dispensing data may differ substantially in their clinical meaning. A change between products containing the same active substance may reflect generic substitution, formulation changes, pharmacy‐level substitution, or reimbursement‐related replacement rather than a deliberate change in pharmacological strategy. By contrast, a switch to a different active substance is more likely to represent a clinically meaningful modification of treatment. Distinguishing between within‐substance product switching and active‐ingredient (ATC substance) switching may therefore improve interpretation of treatment trajectories in prescription registry data.

In the Norwegian setting, this distinction is particularly relevant because multiple methylphenidate products and generics are available, and prescribing commonly involves shared care between specialist services and general practitioners. In addition, the prescriber specialty recorded on the last dispensation before non‐persistence may help contextualise the dispensing pathway immediately preceding treatment discontinuity.

### 1.2 Aim

Using nationwide dispensing data, we aimed to provide a detailed longitudinal description of treatment trajectories based on ADHD medication dispensing patterns in Norway after treatment initiation. Specifically, we examined sex‐ and age‐specific persistence, characterised medication switching by distinguishing within‐substance product switching from active‐ingredient (ATC substance) switching, and described the last dispensed medication and prescriber specialty recorded before non‐persistence.

## 2 Methods

### 2.1 Study Design and Data Source

This was a retrospective observational study using data from the Norwegian Prescription Database (NorPD), which records all dispensed prescription drugs in Norwegian pharmacies since 2004. (Drugs dispensed at hospitals, nursing homes, or over the counter are not included.) Ethical approval was obtained from the Regional Committee for Medical and Health Research Ethics, REC Oslo (Approval #344237).

Data were obtained from NorPD via the Norwegian Medicines Agency (Reseptregisteret.no) and processed by Farmastat/Signum Life Science. NorPD contains pseudonymised person‐level records including sex, age, and dispensed medication (ATC code and product/brand) but excludes clinical diagnoses. For this study, Farmastat/Signum generated aggregated incidence measures using linkage across years via the encrypted person identifier within NorPD, but no person‐level data were available to the authors.

### 2.2 Study Population

We identified new users of ADHD medication by selecting individuals with a first dispensed prescription between 1 January 2012 and 31 December 2016. Incident use was defined using an individual‐specific 5‐year look‐back: individuals were included if they had their first dispensed ADHD medication between 1 January 2012 and 31 December 2016 and no dispensations of ADHD medication in the 5 years preceding the index date. The cohort start in 2012 ensured the availability of a complete 5‐year look‐back from 2007 onward. Eligible participants were aged ≥ 6 years at first dispensation; no upper age limit was applied. Because age was supplied to the authors only in aggregated categories, with the oldest category defined as ≥ 28 years, the maximum age in the cohort was not available. Baseline age and sex were recorded at initiation, and participants were categorised into four age groups: 6–11, 12–17, 18–27, and ≥ 28 years. These age groups were prespecified before the aggregated data were obtained. In total, 33 706 new incident users were observed within years 2012–2016 and therefore included in the study.

### 2.3 Follow‐Up and Outcome Measures

Individuals were followed from the index dispensation for up to 5 years. The register observation period ended on 31 December 2021. For discontinuation analyses based on a ≥ 396‐day dispensing gap, classification requires ≥ 396 days of subsequent observation (“look‐ahead”); therefore, time‐at‐risk for discontinuation was administratively truncated on 30 November 2020 (396 days before the register end date).

The following Anatomical Therapeutic Chemical (ATC) codes were included:

N06BA02 (dexamphetamine), N06BA04 (methylphenidate), N06BA07 (modafinil), N06BA09 (atomoxetine), N06BA12 (lisdexamphetamine), and C02AC02 (guanfacine).

Dispensations of other ATC code medications observed in raw switching sequences (e.g., modafinil) were excluded from the primary switching analyses and are reported only in Table S2. Clonidine was not included.

Treatment discontinuation (non‐persistence) was operationalised using dispensing gaps. For each individual, dispensations of ADHD medication were ordered chronologically from the index date. The longest possible medicine prescription issued in Norway is limited to a maximum of 12 months. A treatment episode was therefore considered ongoing as long as the time between consecutive dispensations was < 396 days (13 months). Discontinuation was defined as the first occurrence of a dispensing gap of ≥ 396 days. Under this approach, the discontinuation date was assigned to the dispensing date of the last prescription preceding the ≥ 396‐day gap (i.e., the last observed dispensation within the treatment episode). Participants without a qualifying gap before the end of follow‐up were censored without meeting the non‐persistence definition; we use this description rather than the term ‘stayers’.

Treatment length was defined as the time from initiation (index dispensation) to the date of discontinuation (as defined above) or censoring. Switches between medications were analysed as two distinct outcomes: (1) within‐substance product switching, defined as a change in the dispensed product (generic or brand name) while the active substance remained unchanged (same ATC code), capturing both brand to generic changes (e.g., due to marketing/reimbursement strategies) and formulation changes within the same substance (e.g., immediate to extended release formulations to increase duration), and (2) active‐substance shifting, defined as a change in active ingredient (ATC substance) between consecutive dispensations within ongoing treatment period.

The number of switches per patient during the first 5 years was classified as 0, 1, 2, 3, or ≥ 4. Dose changes within the same brand were not counted as switches.

Last prescriber specialty was categorised as (1) general practitioner or (2) psychiatrist/another specialist. In Norway, only child and adolescent psychiatrists, paediatricians, adult psychiatrists, and neurologists may initiate medication with central stimulants, whereas general practitioners (GPs) may continue treatment based on a specialist's recommendation. This practice is similar in Denmark and Finland, but not in Sweden where prescribing and renewals of stimulants occur within specialist care.

### 2.4 Statistical Analysis

Baseline characteristics were summarised using counts and percentages for categorical variables and means (standard deviations) or medians (interquartile ranges) for continuous variables, as appropriate. Primary outcomes were described overall and stratified by sex and age group.

Persistence of dispensing ADHD medication was estimated using Kaplan–Meier methods, with time zero defined as the index dispensation. Discontinuation (non‐persistence) was treated as the event and defined by the first dispensing gap of ≥ 396 days, the event time was assigned to the date of the last dispensation preceding the qualifying gap. Individuals were censored at the earliest of (i) 5 years after initiation, (ii) death/emigration if available in the dataset (otherwise not applicable), or (iii) administrative censoring required for gap‐based classification. Because identifying a ≥ 396‐day gap requires look‐ahead, discontinuation analyses were administratively censored 396 days before the register end date (i.e., 30 November 2020 for a register end date of 31 December 2021). We report survival probabilities at prespecified time points (every 28 days) with 95% confidence intervals (Greenwood's method) and the median time to discontinuation were estimable. Kaplan–Meier curves are presented stratified by sex and by age group within sex.

Medication switching was summarised as (i) the number of switches during follow‐up (0, 1, 2, 3, or ≥ 4) and (ii) the type of first switch (within‐substance product switch vs. active‐ingredient switch), stratified by sex and age group. All analyses were conducted by Signum on anonymised registry data using IBM SPSS Statistics (IBM Corp., Armonk, NY, USA); statistical tests were not the primary focus and are reported only where relevant.

## 3 Results

### 3.1 Study Population

Among 33 706 new ADHD medicine users, 13 808 (41%) women and 19 898 (59%) men, the mean age at medical treatment initiation was 23.0 years (SD = 14.3). The reported age groups at initiation were: 6–11 years, 9475 (28.1%); 12–17 years, 6432 (19.1%); 18–27 years, 6784 (20.1%); and ≥ 28 years, 10 878 (32.3%). Age strata were mutually exclusive and were assigned from age at treatment initiation.

### 3.2 Treatment Persistence and Time to Non‐Persistence

Kaplan–Meier estimates of dispensing‐based persistence are shown by sex in Figure 1 and summarised at selected time points in Table 1. The median treatment length was 2.5 years for females and 3.0 years for males. After 5 years, 35% of females and 39% of males remained on treatment. Overall, 21 041 (62.4%) met the dispensing‐based non‐persistence definition during follow‐up, while 12 665 (37.6%) were censored without meeting the definition indicating still on pharmacological treatment at 5 years. At all selected time points, Kaplan–Meier persistence estimates were higher in males than in females; approximate pointwise comparisons suggested statistically significant differences throughout follow‐up (all *p* < 0.01). Age‐stratified persistence curves are shown separately for males and females in Figure 2A,B, where the youngest age group 6–11 years had longer treatment persistence compared to adults.

**FIGURE 1:**
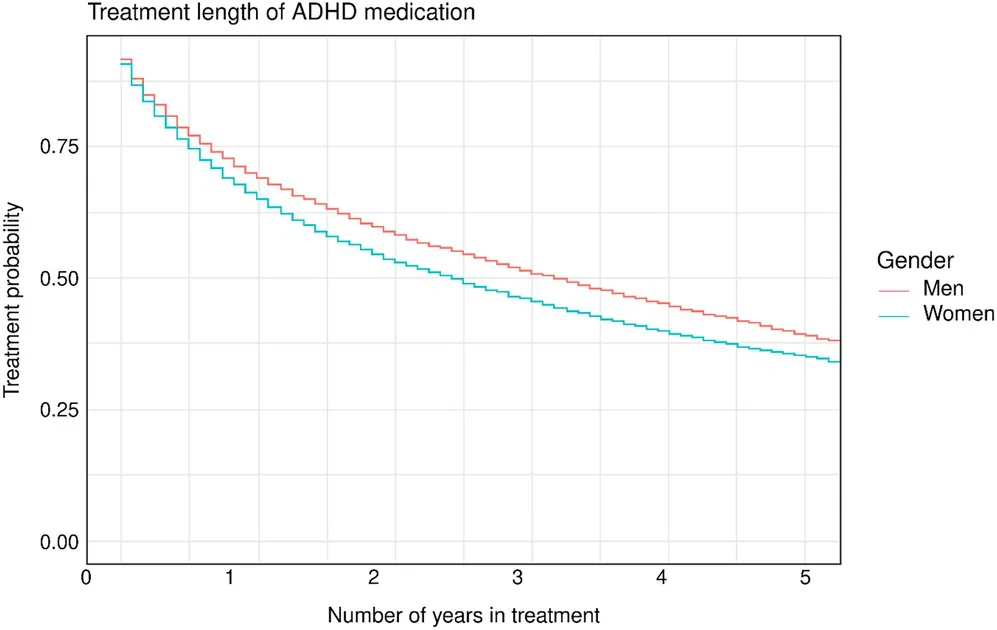
Shows a “Kaplan‐Meier survival analysis” of treatment drop‐out stratified by gender. Women drop out of their medical treatment to a greater extent compared to men. After 5 years of continuous treatment ca. 38% of men are still in treatment, and ca. 34% of women are in treatment.

**TABLE 1:** Kaplan–Meier persistence estimates at selected time points, stratified by sex, among incident ADHD medication users (2012–2016).

Survival time	At risk	Estimate	Standard error	95% CI
Survival statistics: Male				
At start	19 898	1.000		
28 days	18 104	0.913	0.002	[0.91–0.92]
1 year	13 851	0.690	0.005	[0.68–0.70]
2 years	11 630	0.583	0.006	[0.58–0.59]
3 years	10 155	0.511	0.007	[0.51–0.52]
4 years	8917	0.445	0.008	[0.44–0.45]
5 years	7713	0.389	0.009	[0.38–0.40]
Survival statistics: Female				
At start	13 808	1.000		
28 days	12 474	0.903	0.003	[0.90–0.91]
1 year	9114	0.652	0.006	[0.64–0.66]
2 years	7387	0.533	0.008	[0.53–0.54]
3 years	6327	0.457	0.009	[0.45–0.47]
4 years	5491	0.393	0.011	[0.39–0.40]
5 years	4815	0.349	0.012	[0.34–0.36]

*Note:* Discontinuation was defined as the first dispensing gap ≥ 396 days (13 months), with the event dated to the last dispensation preceding that gap. Participants were censored at the earliest of 5 years after initiation.

**FIGURE 2:**
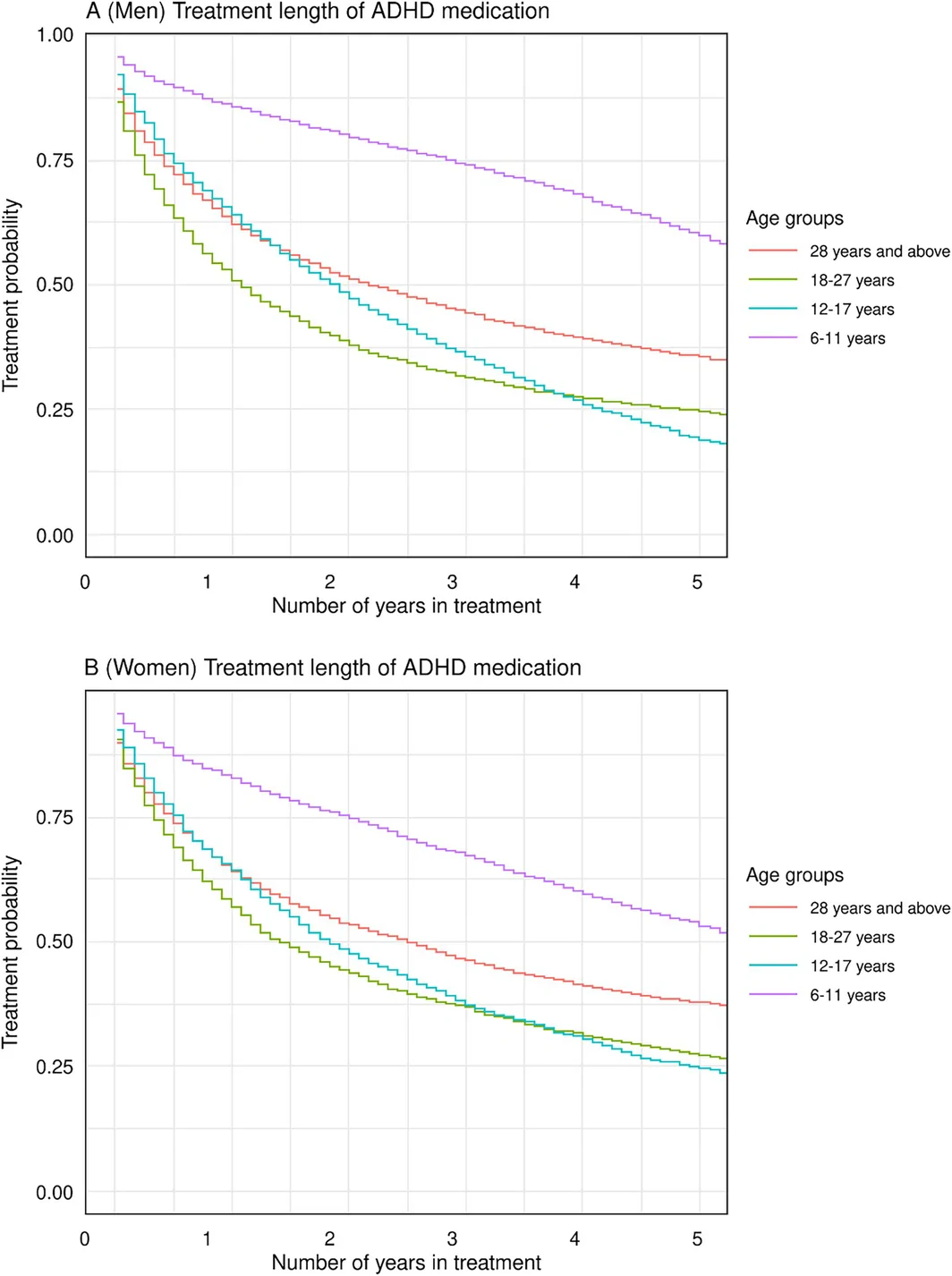
(A, B) Shows Kaplan‐Meier survival analysis of the treatment length of 13 808 women (Figure 3A) and 19 898 men (Figure 3B) stratified by four age groups. The drop‐out analysis is based on New Norwegian incident patients in the period 2012–2016. All patients were followed 6 years from their initiation of ADHD medication and drop‐out is registered when a patient had a break in ADHD medicine redemptions of 13 months or more.

### 3.3 Switching Frequency

Methylphenidate was the initial medication for the vast majority, 98% of females and 97% of males. The remaining users started with atomoxetine.

The number of overall medication switches (i.e., within and between active substances) during follow‐up was: 41% had no switch, 15% had one, 12% had two, 7% had three, and 26% had four or more switches. Each participant was assigned to one mutually exclusive switch‐count category. In Table 2, the sex‐ and age‐specific rows are nested within the non‐persistent group and are not additional participant groups. No major sex differences were observed (Table 2).

**TABLE 2:** Number of ADHD medication product switches during follow‐up by persistence status, sex, and age at initiation.

Group	Participants	No switch	1 switch	2 switches	3 switches	≥ 4 switches
Full cohort	33 706	13 789 (41%)	4923 (15%)	3985 (12%)	2398 (7%)	8611 (26%)
Met non‐persistence definition	21 041	8766 (42%)	3796 (18%)	2653 (13%)	1536 (7.3%)	4290 (20%)
Females	8968	3676 (41%)	1682 (19%)	1181 (13%)	635 (7%)	1794 (20%)
Girls, 6–11 years	1195	326 (27%)	190 (16%)	195 (16%)	109 (9%)	375 (31%)
Girls, 12–17 years	1991	618 (31%)	394 (20%)	321 (16%)	181 (9%)	477 (24%)
Women, 18–27 years	2543	1164 (46%)	522 (21%)	322 (13%)	151 (6%)	384 (15%)
Women, ≥ 28 years	3239	1568 (48%)	576 (18%)	343 (11%)	194 (6%)	558 (17%)
Males	12 073	5090 (42%)	2114 (17.5%)	1472 (12%)	901 (7.5%)	2496 (21%)
Boys, 6–11 years	2793	725 (26%)	471 (17%)	400 (14%)	269 (9.6%)	928 (33%)
Boys, 12–17 years	3088	1069 (35%)	550 (18%)	485 (16%)	282 (9%)	702 (23%)
Men, 18–27 years	2501	1346 (54%)	468 (19%)	254 (10%)	147 (6%)	286 (11%)
Men, ≥ 28 years	3691	1950 (53%)	625 (17%)	333 (9%)	203 (5%)	580 (16%)
Censored^a^	12 665	5023 (40%)	1127 (9%)	1332 (11%)	862 (7%)	4321 (34%)

*Note:* Values are *n* (%) within each row. A switch was recorded when a participant redeemed a different ADHD medication product, either within or between active substances, during an ongoing treatment episode (consecutive dispensations separated by < 396 days). The full cohort comprises participants who met the non‐persistence definition and participants censored without meeting it. Sex‐ and age‐specific rows are nested within the non‐persistent group; participants entered only one age stratum and one switch‐count category.

^a^ Censored without meeting the non‐persistence definition. Counts reported as < 5 were replaced by 2 for calculation of percentages. Minor substances/products not shown may cause row sums to be lower than the number of participants.

When switching to a second medication, 71%–76% changed to another methylphenidate formulation, 9%–11% to atomoxetine, 9%–11% to lisdexamphetamine, and 4%–5% to dexamphetamine. The distribution by sex of first product switches is illustrated in Figure 3A (females) and Figure 3B (males), showing that most first switches originated from methylphenidate products and most second choices were another methylphenidate formulation.

**FIGURE 3:**
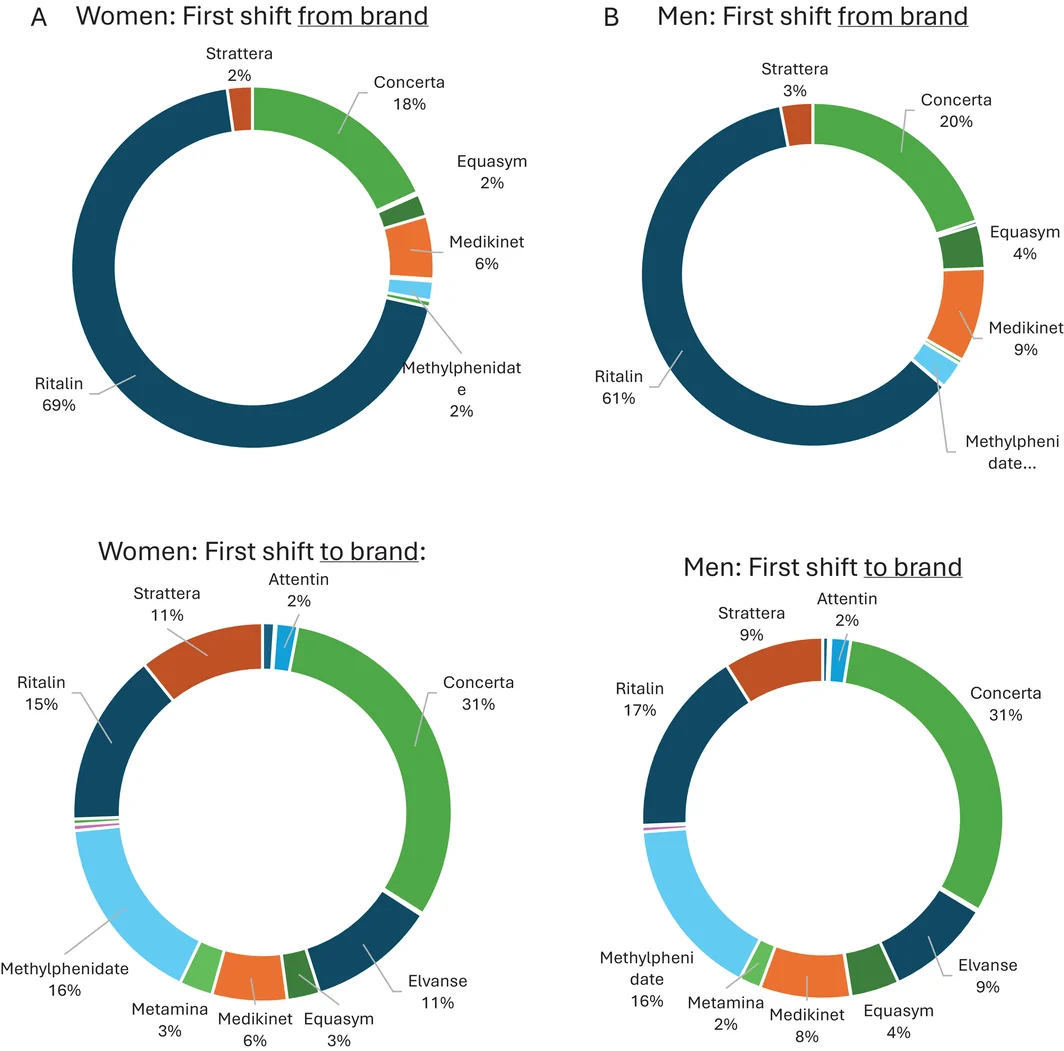
(A) Shows Female incident patients ‐ first shift in medication. The chart is based on female incident patients in the period 2012–2016. The chart displays the first shift between brands. Only patients registered with minimum one shift within 5 years after initiation of ADHD medication is included in the charts. A shift is registered when a patient redeems another brand within a period of 13 months. Female patients show 69% percent shift from Ritalin, and 18% percent shift from Concerta. Concerta, Ritalin and Methylphenidate stands for more than 60% of second choices. (B) Shows Male Incident patients: first shift of medication: **Definitions: The chart displays the first shift between brands. Only patients registered with minimum one shift 5 years after initiation of ADHD medication is included in the charts. A shift is registered when a patient redeems another brand within a period of 13 months. 61% percent of men registered with a first shift in medication shift from Ritalin, and 20% percent shift from Concerta. Together, Concerta, Ritalin and Methylphenidate stand for more than 60% of second choices.

Among individuals with ≥ 1 switch during follow‐up, the first switch was predominantly a within‐substance product switch (same ATC substance): 80.9% in males and 77.5% in females (Table 3). Among within‐substance first switches, transitions were dominated by switches between methylphenidate products (see Table S1).

**TABLE 3:** Type of first ADHD medication switch within 5 years, by sex and age at initiation.

Sex	Age group	Within‐substance product switches (same ATC)	Active‐ingredient switches (ATC changes)	Total first switches
Female	6–11	1842 (86.6%)	285 (13.4%)	2127
Female	12–17	1661 (83.6%)	327 (16.4%)	1988
Female	18–27	1648 (73.2%)	603 (26.8%)	2251
Female	28+	2418 (71.1%)	982 (28.9%)	3400
Female	All	7569 (77.5%)	2197 (22.5%)	9766
Male	6–11	5273 (88.2%)	705 (11.8%)	5978
Male	12–17	2295 (85.7%)	382 (14.3%)	2677
Male	18–27	1398 (72.2%)	538 (27.8%)	1936
Male	28+	2462 (69.7%)	1069 (30.3%)	3531
Male	All	11 428 (80.9%)	2694 (19.1%)	14 122
Both	All	18 997 (79.5%)	4891 (20.5%)	23 888

*Note:* The analysis includes incident users (2012–2016) with ≥ 1 switch during follow‐up. A switch was defined as a change in the dispensed ADHD medication product during an ongoing treatment episode (consecutive dispensations separated by < 396 days). First switches were classified as within‐substance product switches (unchanged ATC substance; brand/generic and/or formulation change) or active‐ingredient switches (change in ATC substance). Values are *n* (%), with percentages calculated within each sex × age‐group stratum. Counts reported as < 5 were replaced by 2 for calculation of percentages.

Switch between different substance products (different ATC codes) was 19.1% for males and 22.5% for females and increasing with increasing age for both sexes (Table 3). Aggregated switches at the ATC substance level, the most common active‐ingredient transitions were methylphenidate → lisdexamfetamine and methylphenidate → atomoxetine, together accounting for approximately two‐thirds of ATC‐changing first switches (Table 4). Among active‐ingredient (ATC‐changing) switches, the two most common pathways included methylphenidate to lisdexamfetamine, and methylphenidate to atomoxetine. (See Table S2).

**TABLE 4:** Most common ATC substance transitions among active‐ingredient first switches, by sex.

From (ATC substance)	To (ATC substance)	Overall *n* (%)	Men *n* (%)	Women *n* (%)
Methylphenidate	Lisdexamfetamine	1510 (32.4)	827 (31.8)	683 (33.2)
Methylphenidate	Atomoxetine	1471 (31.6)	800 (30.7)	671 (32.6)
Atomoxetine	Methylphenidate	655 (14.1)	427 (16.4)	228 (11.1)
Methylphenidate	Dexamphetamine	619 (13.3)	323 (12.4)	296 (14.4)
Lisdexamfetamine	Methylphenidate	120 (2.6)	66 (2.5)	54 (2.6)
Dexamphetamine	Methylphenidate	50 (1.1)	28 (1.1)	22 (1.1)
Lisdexamfetamine	Dexamphetamine	49 (1.1)	25 (1.0)	24 (1.2)
Atomoxetine	Lisdexamfetamine	44 (0.9)	24 (0.9)	20 (1.0)

*Note:* Values are *n* (% of all active‐ingredient first switches) within each column. Percentages are calculated among active‐ingredient switches only. Counts reported as < 5 were replaced by 2 for calculation of percentages. Transitions to or from modafinil were excluded from the primary ADHD medication list.

### 3.4 Dispensing Context Preceding Non‐Persistence

Among the 62% who discontinued treatment, the last dispensed ADHD medication preceding non‐persistence was most often methylphenidate (76%), followed by atomoxetine/guanfacine (12%), lisdexamfetamine (7%), and dexamphetamine (3%) (Table 5).

**TABLE 5:** Last dispensed ADHD medication before non‐persistence, by sex and age at initiation.

Group	Participants	Methylphenidate	Atomoxetine/Guanfacine	Lisdexamfetamine	Dexamphetamine
Girls 6–11	1195	877	178	125	5
Girls 12–17	1991	1574	193	195	10
Women 18–27	2543	1998	276	126	82
Women 28+	3239	2357	336	207	193
Females	8968	6806 (76%)	983 (11%)	653 (7%)	290 (3%)
Boys 6–11	2793	2107	395	263	21
Boys 12–17	3088	2540	299	220	9
Men 18–27	2501	1915	305	148	89
Men 28+	3691	2512	509	249	284
Males	12 073	9074 (75%)	1508 (12%)	880 (7%)	403 (3%)
All non‐persistent participants	21 041	15 880 (76%)	2491 (12%)	1533 (7%)	693 (3%)

*Note:* The analysis includes only participants who met the non‐persistence definition. Values are n; percentages in total rows use the number of non‐persistent participants in that row as denominator. Counts reported as < 5 were replaced by 2 for calculation of percentages. Minor substances/products not shown may cause row sums to be lower than the number of participants.

The last prescriber specialty recorded on the last dispensation was a general practitioner (GP) for 51.9% and a specialist for 48.1%, with a higher proportion of GP prescribers in older age groups (Table 6).

**TABLE 6:** Prescriber specialty recorded at the last dispensation before non‐persistence, by age at initiation.

Age group	Participants	General practitioner, *n* (%)	Specialist *n* (%)
6–11 years	3988	2370 (59.4)	1618 (40.6)
12–17 years	5079	2841 (56.0)	2238 (44.0)
18–27 years	5044	2419 (48.0)	2625 (52.0)
28+ years	6930	3291 (47.5)	3639 (52.5)
All non‐persistent participants	21 041	10 921 (51.9)	10 120 (48.1)

*Note:* The analysis includes only participants who met the non‐persistence definition. Prescriber specialty was categorised as (1) general practitioner and (2) specialist (child and adolescent psychiatrist, paediatrician, adult psychiatrist, or neurologist, as recorded in NorPD). Values are *n* (% within age group).

## 4 Discussion

In this large national register‐based cohort study of incident ADHD medication users in Norway (2012–2016), we characterised three complementary aspects of pharmacotherapy: (1) long‐term persistence and time to discontinuation after initiation, (2) switching dynamics during ongoing treatment by distinguishing within‐substance product switching from active‐ingredient (ATC substance) switching and mapping common first‐to‐second transition pathways, and (3) the dispensing context immediately preceding non‐persistence, captured by the last dispensed medication and prescriber specialty recorded on the last dispensation.

We observed near‐universal initiation on methylphenidate and modest five‐year persistence (39% in men and 35% in women). Switching was common; approximately one quarter of patients underwent four or more medication switches. The first switches were predominantly within‐substance products for approximately four‐fifths of the patients. Among discontinuers, methylphenidate was the last dispensed medication for 76%. The last recorded prescriber was a GP for just over half of the patients.

These dispensing patterns correspond well with prior internationally reported trends, including the Norway‐specific persistence estimates reported by Brikell et al. [8], and extend the literature by adding a more granular description of product‐level switching and the prescribing context around the last observed dispensation. Because registry data from NorPD captures dispensations rather than clinical decision‐making or medication ingestion, the findings should only be interpreted as descriptions of routine care.

### 4.1 Treatment Persistence and Discontinuation

More than 60% stopped retrieving medication within 5 years, consistent with prior studies showing rapid decline in ADHD treatment continuity [8]. Adolescents and young adults showed the shortest treatment duration, and this pattern may reflect multiple factors, both related to the individual and to the health care system (e.g., organisation of follow‐up during the transition from specialist to primary care). Potential individual explanations include reduced symptom burden, life transitions/changed social context, acquired better coping strategies, perceived adverse effects, and varying attitudes toward long‐term medication, which are commonly reported drivers of discontinuation [9]. Taken together, these patterns raise the hypothesis that persistence is shaped by a combination of developmental transitions and follow‐up needs that may vary by age and sex and could be evaluated in studies linking dispensations to clinical contacts and treatment intentions.

### 4.2 Medication Switching and Brand Variation

A notable feature of the Norwegian context is the large number of available methylphenidate brands and generics. Frequent switching between products may reflect factors such as pharmacy‐level substitution practices, reimbursement policies, or prescriber preferences, rather than genuine differences in clinical efficacy. Brand confusion could also contribute to discontinuation if patients perceive such changes as instability or inconsistency in treatment. Figure 3A,B illustrate that early switching was overwhelmingly from Ritalin and Concerta, supporting the interpretation that pharmacy substitution or formulation tolerability rather than lack of efficacy may drive early changes. The introduction of multiple generics during the study period likely contributed to the high rate of brand switching, especially visible in the first‐shift data (Figure 3A,B). Previous Norwegian clinical studies suggest that changes in ADHD medication over time may reflect clinically driven treatment adjustments related to effectiveness, tolerability, and adherence. At the same time, product‐level changes in dispensing data may also reflect pharmacy substitution or formulation changes, which can appear as “switching” without representing a change in pharmacological strategy [13, 14].

### 4.3 Prescriber Role and Continuity of Care

About half of discontinuers had a general practitioner recorded as the professional specialty on the last dispensed prescription before discontinuation (Table 6). In Norway, GPs are permitted to continue, but not to initiate or modify stimulant prescriptions. Such structural limitation may hinder dosage optimisation or re‐initiation after interruption. Closer collaboration between GPs and psychiatrists, along with national guidance on shared care, could help sustain adherence. However, this measure reflects only the prescriber's specialty recorded in the dispensing register and does not capture consultations, duration of treatment, shared‐care arrangements, or specialist involvement that did not result in a dispensation. The findings should therefore be interpreted with caution, as they describe the prescribing context at the last observed dispensation rather than indicating the absence of adequate follow‐up; specialist involvement, including from mental health care, may still have been present. This may also indicate that an initial treatment trial under specialist care was not effective, or that the transition to general practitioner‐led care did not function as intended.

We also acknowledge that discontinuation of ADHD medication may be clinically appropriate in some cases, and even a long‐term goal, where coping strategies and changes of life context have been part of a treatment plan. Evidence from the Multimodal Treatment Study of ADHD (MTA) indicates that symptoms and associated functional impairments can fluctuate over time, and that treatment needs may consequently change, with the benefits of continuous medication attenuating in some individuals. Accordingly, treatment duration should be individualised and regularly re‐evaluated.

Future studies linking dispensing records with clinical contact data may provide a more comprehensive understanding of care pathways surrounding discontinuation. The present findings may suggest that the organisation of shared‐care pathways, such as communication between psychiatrists and general practitioners, and the frequency of follow‐up at the point of transfer could influence continuity of medication dispensing. This warrants further investigation using linked data on prescriber transitions and clinical follow‐up.

### 4.4 Clinical and Policy Implications

This study identified challenges in treatment continuity, particularly during adolescence and young adulthood. Since our registry data reflects dispensing patterns rather than clinical outcomes, these results describe trends rather than an explanation of treatment trajectories.

However, these findings may help identify periods where treatment continuity is more challenging, particularly during adolescence and young adulthood. Evidence syntheses have highlighted that adherence and persistence are influenced by several modifiable factors, and that structured interventions may improve continuity in certain settings [10, 15, 16]. Because NorPD does not capture clinical indication, symptom course, reasons for changes, or clinical outcomes, our results should be interpreted as descriptive of dispensing‐based patterns rather than as evidence for causal mechanisms.

Nevertheless, the predominance of within‐substance product switching (unchanged ATC substance) suggests that many observed switches are unlikely to represent a deliberate choice of a “better” medicine and may instead reflect generic substitution, price/reimbursement incentives, and pharmacy‐level substitution practices. Some active‐ingredient switches may also be influenced by perceived loss of effect or tolerability over time [17]. Clear information at prescription and structured follow‐up during care transitions could be evaluated as potential strategies to support persistence when continued medication is clinically indicated [10].

Taken together, these observations raise the hypothesis that reimbursement and substitution policies interact with patient perceptions (e.g., possible confusion about changing product names/formulations) to shape observed switching trajectories and, potentially, dispensing continuity; this could be tested in future studies linking dispensations to policy changes, pharmacy substitution records, and patient‐reported experiences.

### 4.5 Strengths and Limitations

The main strengths of this study include nationwide coverage, long follow‐up, and inclusion of incident users. A main limitation is that data from NorPD is dispensing‐based and does not include clinical data (e.g., symptom severity, comorbidity, compliance, side effects, lack of effect, or losing effect.) and lack ability to confirm ingestion or adherence. Brand‐level switching may overestimate clinically meaningful changes, as generic substitution is common in Norway. The registry lacks dosage information, preventing distinction between titration, optimisation, and brand changes. Furthermore, the 13‐month gap criterion classifies treatment interruptions shorter than 396 days as continuation; it may therefore overestimate persistence and underestimate non‐persistence when shorter temporary breaks occur.

Comparisons of findings with other countries, including neighbouring Nordic nations, should be interpreted with caution, as switching patterns may be influenced by differences in regulatory frameworks, marketing practices, and clinical traditions influencing the prescribing of stimulants and other ADHD medications.

Finally, the cohort in this study is from 2012 to 2016 with 5 years follow up until 2021. The increased prevalence of ADHD and medication since then, especially among adults, changes in diagnostic criteria, and introduction of new medication formulas (e.g., of lisdexamfetamine and guanfacine) may limit the direct transferability to more recent cohorts.

## 5 Conclusions

Methylphenidate remains the overwhelmingly dominant first‐line ADHD medication in Norway. However, only about one‐third of users remain on continuous treatment after 5 years. High rates of medication switching and early discontinuation, especially among younger patients, suggest that continuity of follow‐up and coordination between specialist and primary care services warrant further attention.

## Funding

Medice Norway made the statistical material available for this paper, but had no role in study design, analysis, or interpretation of the material.

## Ethics Statement

Approved by the Regional Committee for Medical and Health Research Ethics (REK Oslo #344237). Individual consent was waived due to anonymised data.

## Conflicts of Interest

EA received travel and accommodation support from Medice to attend international conferences. The other authors declare no conflicts of interest.

## Supporting information


**Table S1:** Ten most frequent brand name switches within‐substance first‐to‐second product switch (all are methylphenidate—unchanged ATC substance), stratified by sex. Values are *n* (% of all within‐substance first switches) within each column (Overall/Male/Female). Percentages are calculated within‐substance switches only.
**Table S2:** Ten most frequent brand name switches. First‐to‐second medication transitions where the ATC substance changed (active‐ingredient switches), stratified by sex. Values are *n* (% of all active‐ingredient first switches) within each column (Overall/Men/Women). Percentages are calculated within active‐ingredient switches only.

## Data Availability

Raw data were generated by the Norwegian Prescription Database (NorPD). Derived data supporting the findings are available from the corresponding author upon request.

## References

[1] American Psychiatric Association , ed., Diagnostic and Statistical Manual of Mental Disorders, 5th ed. (American Psychiatric Association, 2013), 10.1176/appi.books.9780890425596.

[2] S. V. Faraone , M. A. Bellgrove , I. Brikell , et al., “Attention‐Deficit/Hyperactivity Disorder,” Nature Reviews. Disease Primers 10, no. 1 (2024): 11, 10.1038/s41572-024-00495-0. 38388701

[3] S. Cortese , N. Adamo , C. Del Giovane , et al., “Comparative Efficacy and Tolerability of Medications for Attention‐Deficit/Hyperactivity Disorder in Children, Adolescents, and Adults: A Systematic Review and Network Meta‐Analysis,” Lancet Psychiatry 5, no. 9 (2018): 727–738, 10.1016/S2215-0366(18)30269-4. PMC6109107 30097390

[4] M. Fredriksen , A. Halmøy , S. V. Faraone , and J. Haavik , “Long‐Term Efficacy and Safety of Treatment With Stimulants and Atomoxetine in Adult ADHD: A Review of Controlled and Naturalistic Studies,” European Neuropsychopharmacology 23, no. 6 (2013): 508–527, 10.1016/j.euroneuro.2012.07.016. 22917983

[5] A. M. S. Sørensen , R. Wesselhöeft , J. H. Andersen , et al., “Trends in Use of Attention Deficit Hyperactivity Disorder Medication Among Children and Adolescents in Scandinavia in 2010–2020,” European Child & Adolescent Psychiatry 32, no. 10 (2023): 2049–2056, 10.1007/s00787-022-02034-2. 35831669

[6] S. R. Raman , K. K. C. Man , S. Bahmanyar , et al., “Trends in Attention‐Deficit Hyperactivity Disorder Medication Use: A Retrospective Observational Study Using Population‐Based Databases,” Lancet Psychiatry 5, no. 10 (2018): 824–835, 10.1016/S2215-0366(18)30293-1. 30220514

[7] I. Hartz , N. H. H. Madsstuen , P. N. Andersen , M. Handal , and I. Odsbu , “Nationwide Trends in the Use of ADHD Medications in the Period 2006–2022: A Study From the Norwegian Prescription Database,” BMC Psychiatry 24, no. 1 (2024): 767, 10.1186/s12888-024-06199-9. PMC11539614 39501180

[8] I. Brikell , H. Yao , L. Li , et al., “ADHD Medication Discontinuation and Persistence Across the Lifespan: A Retrospective Observational Study Using Population‐Based Databases,” Lancet Psychiatry 11, no. 1 (2024): 16–26, 10.1016/S2215-0366(23)00332-2. 38035876

[9] K. Gajria , V. Sikirica , M. Copley‐MacDougall , et al., “Adherence, Persistence, and Medication Discontinuation in Patients With Attention‐Deficit/Hyperactivity Disorder: A Systematic Literature Review,” Neuropsychiatric Disease and Treatment 10 (2014): 1543–1569, 10.2147/NDT.S65721. PMC4149449 25187718

[10] R. Parkin , F. McNicholas , and J. C. Hayden , “A Systematic Review of Interventions to Enhance Adherence and Persistence With ADHD Pharmacotherapy,” Journal of Psychiatric Research 152 (2022): 201–218, 10.1016/j.jpsychires.2022.05.044. 35753240

[11] M. B. Giacobini , J. Zhao , J. Freilich , C. Malmgren , N. Wallin Bernhardsson , and E. Ahnemark , “Transitioning From Pediatric to Adult Psychiatric Care for ADHD in Sweden: A Nationwide Study,” Journal of Attention Disorders 29, no. 4 (2025): 235–243, 10.1177/10870547241303259. PMC11697494 39720843

[12] Helsedirektoratet , “ADHD/hyperkinetisk forstyrrelse: nasjonal faglig retningslinje for utredning, behandling og oppfølging,” (2021), https://www.helsedirektoratet.no/retningslinjer/adhd‐hyperkinetisk‐forstyrrelse.

[13] M. Fredriksen , A. A. Dahl , E. W. Martinsen , O. Klungsøyr , J. Haavik , and D. E. Peleikis , “Effectiveness of One‐Year Pharmacological Treatment of Adult Attention‐Deficit/Hyperactivity Disorder: An Open‐Label Prospective Study of Time in Treatment, Dose, Side‐Effects and Comorbidity,” European Neuropsychopharmacology 24, no. 12 (2014): 1873–1884, 10.1016/j.euroneuro.2014.09.013. 25453480

[14] M. Fredriksen and D. E. Peleikis , “Long‐Term Pharmacotherapy of Adults With Attention Deficit Hyperactivity Disorder: A Literature Review and Clinical Study,” Basic & Clinical Pharmacology & Toxicology 118, no. 1 (2016): 23–31, 10.1111/bcpt.12477. 26404187

[15] M. Ferrin , A. Häge , J. Swanson , et al., “Medication Adherence and Persistence in Children and Adolescents With Attention Deficit Hyperactivity Disorder (ADHD): A Systematic Review and Qualitative Update,” European Child & Adolescent Psychiatry 34, no. 3 (2025): 867–882, 10.1007/s00787-024-02538-z. 39105823

[16] N. Valaei Sharif , P. Ghasemzadeh , and S. Ghasemzadeh , “Strategies That Enhance Medication Adherence in Children and Adolescents With Attention Deficit Hyperactivity Disorder: Update From the European ADHD Guidelines Group,” Current Developmental Disorders Reports 11, no. 3 (2024): 113–124, 10.1007/s40474-024-00299-7.

[17] X. Castells , C. Blanco , D. Bruno , et al., “Tolerance to Stimulant Medication for Attention‐Deficit/Hyperactivity Disorder: A Review,” Neuropsychiatric Disease and Treatment 19 (2023): 2279–2291, 10.2147/NDT.S394455.

