# Supplementary material for: Long‐Term Treatment Patterns in Norwegian ADHD‐Medicine Users: A Cohort Register‐Based Study With 5 Years of Follow‐Up, 2012–2021
Source: Pharmacoepidemiol Drug Saf. 2026 Oct 12;35(10):e70486. doi: 10.1002/pds.70486 (PMC13657855; doi:10.1002/pds.70486)
Supplement: Supplementary file 1 [file PDS-35-e70486-s001.docx]

**SUPPLEMENTARY MATERIAL**

**Table S1. Ten most frequent brand name switches within-substance first-to-second product switch (all are methylphenidate - unchanged ATC substance), stratified by sex.** Values are n (% of all within-substance first switches) within each column (Overall/Male/Female). Percentages are calculated within within-substance switches only.

| **From (product)** | **To (product)** | **Overall, n (%)** | **Male, n (%)** | **Female, n (%)** |
| --- | --- | --- | --- | --- |
| Ritalin® tab | Ritalin® caps | 4018 (21.2) | 2202 (19.3) | 1816 (24.0) |
| Ritalin® caps | Ritalin® tab | 3132 (16.5) | 1902 (16.6) | 1230 (16.3) |
| Ritalin® caps | Concerta® depottab | 1639 (8.6) | 969 (8.5) | 670 (8.9) |
| Ritalin® tab | Concerta® depottab | 1550 (8.2) | 868 (7.6) | 682 (9.0) |
| Concerta® depottab | Methylphenidate® Sandoz | 1080 (5.7) | 701 (6.1) | 379 (5.0) |
| Concerta® depottab | Ritalin® tab | 968 (5.1) | 573 (5.0) | 395 (5.2) |
| Medikinet® caps | Medikinet® tab | 655 (3.4) | 453 (4.0) | 202 (2.7) |
| Ritalin® caps | Methylphenidate® Teva | 629 (3.3) | 349 (3.1) | 280 (3.7) |
| Concerta® depottab | Ritalin® caps | 594 (3.1) | 377 (3.3) | 217 (2.9) |
| Ritalin® caps | Methylphenidate® Sandoz | 476 (2.5) | 256 (2.2) | 220 (2.9) |

*Footnote:* Counts reported as **<5** were replaced by **2** for calculation of percentages.

**Table S2. Ten most frequent brand name switches. First-to-second medication transitions where the ATC substance changed (active-ingredient switches), stratified by sex.** Values are n (% of all active-ingredient first switches) within each column (Overall/Men/Women). Percentages are calculated within active-ingredient switches only.

| **From (product)** | **To (product)** | **Overall n (%)** | **Men n (%)** | **Women n (%)** |
| --- | --- | --- | --- | --- |
| Ritalin® caps | Elvanse® caps | 937 (19.2) | 496 (18.4) | 441 (20.1) |
| Ritalin® caps | Strattera® caps | 689 (14.1) | 356 (13.2) | 333 (15.2) |
| Strattera® caps | Ritalin® caps | 309 (6.3) | 186 (6.9) | 123 (5.6) |
| Concerta® depottab | Strattera® caps | 305 (6.2) | 185 (6.9) | 120 (5.5) |
| Ritalin® tab | Strattera® caps | 267 (5.5) | 140 (5.2) | 127 (5.8) |
| Ritalin® tab | Metamina® tab | 205 (4.2) | 101 (3.7) | 104 (4.7) |
| Concerta® depottab | Elvanse® caps | 184 (3.8) | 116 (4.3) | 68 (3.1) |
| Ritalin® caps | Attentin® tab | 176 (3.6) | 86 (3.2) | 90 (4.1) |
| Strattera® caps | Ritalin® tab | 163 (3.3) | 108 (4.0) | 55 (2.5) |
| Ritalin® tab | Elvanse® caps | 106 (2.2) | 58 (2.2) | 48 (2.2) |

*Footnote:* Counts reported as **<5** were replaced by **2** for calculation of percentages.
